# Impact of severe intra‐abdominal adhesions on early maternal complications following cesarean delivery

**DOI:** 10.1002/ijgo.16161

**Published:** 2025-01-18

**Authors:** Shai Ram, Hila Shalev‐Ram, Shira Alon, Shai Trigerman, Ariel Many, Yariv Yogev, Emmanuel Attali

**Affiliations:** ^1^ Lis Hospital for Women's Health Tel Aviv Sourasky Medical Center Tel Aviv Israel; ^2^ Faculty of Medicine Tel Aviv University Tel Aviv Israel; ^3^ Department of Obstetrics and Gynecology Meir Medical Center Kfar Saba Israel; ^4^ Department of Statistics and Data Science Hebrew University of Jerusalem Jerusalem Israel

**Keywords:** cesarean delivery complications, intra‐abdominal adhesions, maternal complications

## Abstract

**Objective:**

The rising rates of cesarean delivery (CD), which are a leading cause of intra‐abdominal adhesions, represent a major concern for maternal health. We aimed to describe early maternal complications following CD in women with severe intra‐abdominal adhesions.

**Methods:**

A prospective observational study was conducted at a university‐affiliated tertiary medical center (January 2021 and March 2023) in Israel. Women who underwent CD were assessed for intra‐abdominal adhesions by questionnaires completed by the surgeons. Adhesions were evaluated at four anatomical sites: abdomen‐to‐uterus, uterus‐to‐bladder, skin‐to‐fascia, and other intra‐abdominal sites. Each site was scored on a scale of 0–2, with a total score ranging from 0 to 8. Severe adhesions were defined as a total score ≥5. The primary outcome measures were a composite complication which included at least one or more of postoperative need for blood or iron products, fever, prolonged hospitalization, re‐hospitalization, and emergency room visits.

**Results:**

Overall, 2797 women were included in the study, of whom 295 (10.6%) exhibited severe adhesions. Women with severe adhesions had a higher composite risk for maternal complications following CD (RR = 1.28, 95% confidence interval [CI]: 1.12–1.45, *P* < 0.001), specifically, postoperative need for blood or iron products (RR = 1.71, 95% CI: 1.15–2.55, *P* = 0.007) and prolonged hospitalization (RR = 1.49, 95% CI: 1.10–2.03, *P* = 0.009). There were no significant group differences in the rates of postoperative fever, re‐hospitalization or emergency room visits. In multivariate analysis, severe adhesions had an independent impact on maternal complications (CI: 1.06–1.95, *P* value 0.017).

**Conclusion:**

Severe intra‐abdominal adhesions diagnosed in CD had an impact on early maternal complications.

## Synopsis

Exploring severe intra‐abdominal adhesions impact on early maternal complications post‐cesarean delivery in a large, prospective observational study, revealed a significant impact.

## 1 INTRODUCTION

Postoperative adhesions represent a frequent obstacle in abdominal surgeries.^1^ Cesarean deliveries (CDs) are prominent contributors to the development of intra‐abdominal adhesions among women of childbearing age.^2^ Given the ongoing rise in CD rates, now exceeding 30% in the US,^3^, ^4^ the occurrence of intra‐abdominal adhesions following CD has emerged as a matter of considerable concern. The occurrence of adhesions is closely linked to the number of prior CD, which is a primary risk factor for their formation.^2^, ^5^, ^6^, ^10^, ^14^ Approximately 25%–46% of cases exhibit adhesion formation following the first CD, with rates increasing to 43%–75% after the second CD and reaching as high as 83% after the third CD.^2^, ^5^, ^6^ Intra‐abdominal adhesions associated with CD have been linked to several complications, including bowel obstruction,^2^, ^11^ bladder and urinary tract injuries^2^, ^12^, ^13^ and prolonged delivery time.^5^, ^6^, ^7^ Furthermore, post‐CD adhesions can lead to increased blood transfusion requirements^14^ and serve as a risk factor for various gynecologic issues, such as infertility, an increased risk of ectopic pregnancy,^2^, ^15^, ^16^ chronic pain,^15^ and a rise in the rate of re‐hospitalizations.^8^

Considering the high prevalence of intra‐abdominal adhesions following CD and the resultant risk of substantial maternal complications, together with the scarcity of existing research on this topic, we aimed to delineate the early postoperative complications in women with severe intra‐abdominal adhesions.

## 2 MATERIALS AND METHODS

### 2.1 Study design

This was a prospective observational study designed to assess early maternal post‐CD complications encountered by women with intra‐abdominal adhesions. All consecutive women, for whom the surgeon complete the questionnaires, and underwent a CD between January 2021 and March 2023 at a single university affiliated tertiary center in Israel, were included in the study population. The study was approved by the local institutional review board (0579‐21 TLV).

Adhesions were assessed and recorded by questionnaires that were filled out by surgeons immediately after each CD. This questionnaire is described and validated in our previous study,^17^ and addressed four main anatomical sites: (1) abdomen‐to‐uterus, (2) uterus‐to‐bladder, (3) skin‐to‐fascia, and (4) other (adhesions in the ovarian area, omentum, intestine, or any other intra‐abdominal site). The surgeons were asked to rank the observed adhesions between zero and two: none (score = 0), filmy (score = 1), or dense (score = 2). Filmy adhesions were defined as those that are weak and therefore easily removed without the need of a device, whereas the removal of dense adhesions required scissors, scalpels, or diathermy. The total score for the four sites could range between 0 and 8. The score of 5 and above was considered as a severe degree of adhesions. Women for whom the surgeon did not complete the questionnaires or there was missing information regarding the preoperative, intraoperative, or primary outcome variables, were excluded.

The data that were extracted from electronic medical records included details of maternal medical history, background morbidity, CD, and surgical details. Gestational weight gain (GWG) was determined by computing the difference between the woman's pregestational weight and her weight at the time of admission for the index CD. The duration of surgery was calculated as the time that had elapsed from the initiation of the incision to the conclusion of the operation. Prolonged surgery time was defined as duration surpassing the 90th percentile.

The main outcome measures were risk factors for maternal complications, which encompassed the following criteria: postoperative need for blood or iron products during hospitalization, fever as indicated by a body temperature exceeding 38.0°C during postoperative hospitalization and requiring antibiotic treatment, prolonged hospitalization as defined by postoperative hospital stays exceeding the 90th percentile, re‐hospitalization and emergency room visits within 30 days following hospital discharge. Re‐hospitalization and emergency room visits referred to hospitalization in the gynecology department and visits to the gynecology emergency department, respectively. Composite complication was considered if any one or more of these complications occurred.

### 2.2 Statistical analysis

Descriptive statistics were used to assess the distribution of variables. Continuous variables are summarized as mean values with standard deviations, and categorical variables are summarized as counts and percentages. The comparison between the study groups, women with severe adhesions and those without, was conducted using a Chi‐square for categorical variables and a *t*‐test for continuous variables. The risk ratio (RR) was calculated for each complication separately and for all complications as a composite outcome, comparing the population of women with severe adhesions (score ≥5) to those with non‐severe adhesions (≤4) by a logit model, with 95% confidence interval (CI). Finally, in order to determine whether severe adhesions constitute an independent risk factor for the maternal complications, a univariate analysis was performed for the composite complications. Variables with a *P* value of less than 0.05, were included in a multivariate model to calculate the odds ratio (OR). The analyses were carried out using Python version 3.73. Significance was defined as a *P* value of less than 0.05.

## 3 RESULTS

**FIGURE 1:**
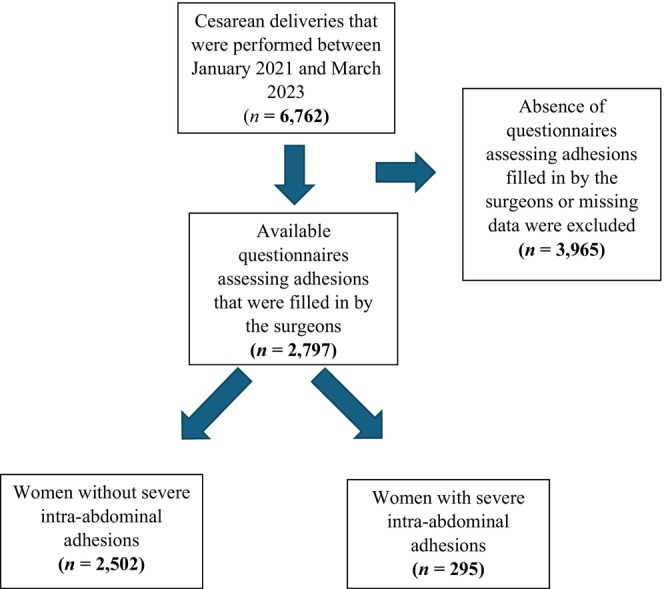
Flow chart of the study participants.

Overall, during the study time, 6762 CDs were performed at our medical center, and the questionnaires assessing for adhesions were filled for 2797 CDs which were included in this study. There were 295 cases (10.6%) of severe adhesions (Figure 1). The characteristics of the study population are presented in Table 1. The mean maternal age in the study group as well as the pre‐pregnancy body mass index (BMI, calculated as weight in kilograms divided by the square of height in meters) were significantly higher compared to women without severe adhesions (35.9 vs. 34.6, *P* < 0.001 and 25.2 vs. 24.1, *P* = 0.003, respectively). There were no significant differences in smoking status, diabetes mellitus, or chronic hypertension between the two groups.

**TABLE 1:** Characteristics of the study populations (*n* = 2797). Demographic and clinical characteristics of women with and without severe intra‐abdominal adhesions.

Variables	Overall	Severe adhesions (score ≥5)		*P* value
		No	Yes	
Number, *n* (%)	2797	2502 (89.4)	295 (10.6)	
Maternal age, years, mean (SD)	**34.7 (5.0)**	**34.6 (5.1)**	**35.9 (4.2)**	**<0.001**
Pre‐pregnancy BMI, mean (SD)	**24.3 (5.7)**	**24.1 (5.7)**	**25.2 (5.6)**	**0.003**
Smoking status, *n* (%)	137 (5.1)	117 (4.9)	20 (6.8)	0.143
Diabetes mellitus, *n* (%)	70 (2.5)	61 (2.4)	9 (3.1)	0.660
Chronic hypertension, *n* (%)	51 (1.8)	43 (1.7)	8 (2.7)	0.329
Adhesion score, mean (SD)	**1.4 (2.1)**	**0.8 (1.3)**	**6.2 (1.1)**	**<0.001**
Previous CD, *n* (%)				
0	**1460 (52.4)**	**1444 (57.9)**	**16 (5.4)**	**<0.001**
1	**886 (31.8)**	**748 (30.0)**	**138 (46.8)**	
2	**350 (12.6)**	**243 (9.7)**	**107 (36.3)**	
3+	**92 (3.2)**	**58 (2.3)**	**33 (11.5)**	
Gestational age at birth, mean (SD)	**38.5 (1.1)**	**38.6 (1.1)**	**38.2 (0.9)**	**<0.001**
Birth weight (grams), mean (SD)	3132 (572)	3129 (570)	3158 (597)	0.429
GWG, mean (SD)	**7.1 (21.7)**	**7.5 (21.5)**	**4.2 (23.5)**	**0.022**
Gestational diabetes mellitus, *n* (%)	586 (21.0)	518 (20.7)	68 (23.1)	0.389
Gestational hypertension, *n* (%)	125 (4.5)	114 (4.6)	11 (3.7)	0.616
ART, *n* (%)	**489 (19.4)**	**452 (19.9)**	**37 (14.4)**	**0.041**
Surgery time, mean (SD)	**34.1 (11.9)**	**33.9 (11.8)**	**35.5 (12.5)**	**0.040**
Prolonged surgery, *n* (%)	**599 (20.4)**	**501 (20.0)**	**77 (26.1)**	**0.041**
Maternal complications, *n* (%)				
Need for blood or iron products	**105 (6.5)**	**84 (5.9)**	**21 (11.0)**	**0.013**
Fever	23 (0.8)	18 (0.7)	5 (1.7)	0.087
Re‐hospitalization	71 (2.5)	64 (2.6)	7 (2.4)	1.000
Prolonged hospitalization	**281 (10.0)**	**239 (9.6)**	**42 (14.2)**	**0.015**
ER visits	306 (10.9)	275 (11.0)	31 (10.5)	0.879

*Note*: BMI, calculated as weight in kilograms divided by the square of height in meters.

Abbreviations: ART, assisted reproductive technology; BMI, body mass index; CD, cesarean delivery; ER, emergency room; GWG, gestational weight gain; SD, standard deviation.

Bold indicates significant.

Other notable group differences included gestational age at birth (38.2 vs. 38.6, *P* < 0.001) and gestational weight gain (4.2 vs. 7.5 kg, respectively, *P* = 0.022). Furthermore, the severe adhesions group had higher rates of prolonged surgery duration (in 26.1% vs. 20.0%, *P* = 0.041) and prolonged hospitalization (14.2% vs. 9.6%, *P* = 0.015), (Table 1). Prolonged surgery duration and prolonged hospitalization, both defined in the methods section as time exceeding the 90th percentile, were found to be more than 79 min for surgery duration and over 4.58 days for hospitalization, respectively.

Table 2 describes the risk ratio for each complication in women with severe intra‐abdominal adhesions compared to those without and the risk for composite complication. Women with severe intra‐abdominal adhesions had a significantly higher need for postoperative blood or iron products administration (RR = 1.71, 95% CI: 1.15–2.55, *P* = 0.007) as well as more prolonged hospitalization (RR = 1.49, 95% CI: 1.10–2.03, *P* = 0.009). The risk for composite complication was also higher in the severe adhesions group (RR = 1.28, 95% CI: 1.12–1.45, *P* < 0.001). There were no significant group differences in re‐hospitalization, emergency room visits, or post‐CD fever. Table 3 describes a multivariate analysis conducted to characterize variables that independently affect maternal complications. It can be observed that even in the multivariate analysis, the most significant factor is severe adhesions (OR = 1.444, CI: 1.06–1.95, *P* value 0.017).

**TABLE 2:** Early maternal post‐cesarean delivery complications in women with severe intra‐abdominal adhesions–relative risk calculation.

Variables	RR	CI 2.5%	CI 97.5%	*P* value
Need for blood or iron products	**1.717**	**1.15**	**2.55**	**0.007**
Prolonged hospitalization	**1.498**	**1.10**	**2.03**	**0.009**
Composite complications	**1.281**	**1.12**	**1.45**	**<0.001**
Re‐hospitalization	0.932	0.43	2.01	0.858
ER visits	0.957	0.67	1.35	0.807
Fever	2.367	0.88	6.32	0.085

Abbreviations: CI, confidence interval; ER, emergency room; RR, risk ratio.

Bold indicates significant.

**TABLE 3:** A multivariate model that describes variables contribution to the composite maternal complications (includes the need for blood or iron products, prolonged hospitalization, re‐hospitalization, ER visits and fever).

Variables	OR	CI 2.5%	CI 97.5%	*P* value
Severe adhesions	**1.444**	**1.06**	**1.95**	**0.017**
Previous CD	0.798	0.60	1.04	0.105
Diabetes mellitus	1.607	0.89	2.87	0.109
Chronic hypertension	1.149	0.60	2.17	0.601
Smoking status	1.092	0.75	1.58	0.641
Pre‐pregnancy BMI	**0.974**	**0.95**	**0.99**	**0.042**
Maternal age	1.013	0.99	1.03	0.145

*Note*: BMI, calculated as weight in kilograms divided by the square of height in meters.

Abbreviations: BMI, body mass index; CD, cesarean delivery; CI, confidence interval; ER, emergency room; OR, odds ratio.

Bold indicates significant.

Table 4 presents the adhesion scores across different anatomical sites observed during cesarean delivery and rated by the surgeons.

**TABLE 4:** Adhesions score across the four anatomical sites, as evaluated by the surgeons.

Adhesions score	Abdomen–uterus	Uterus–bladder	Skin–fascia	Other sites
None–0, *n* (%)	1559 (75.21)	1468 (70.71)	1369 (65.69)	1827 (89.65)
Filmy–1, *n* (%)	296 (14.28)	390 (18.79)	417 (20.01)	141 (6.92)
Dense–2, *n* (%)	218 (10.52)	218 (10.5)	298 (14.3)	70 (3.43)

## 4 DISCUSSION

The findings of the current study revealed a significant association between severe intra‐abdominal adhesions following CD with maternal complications, specifically the need for blood or iron products, and prolonged postoperative hospitalization.

The correlations between the severity of adhesions following CD and maternal complications have not been thoroughly investigated in the existing literature. Most of the reports in the literature refer to maternal complications after repeat CD^5^, ^6^, ^9^, ^10^, ^11^ and not specifically to those associated with the presence or severity of adhesions. However, several reports have correlated severe adhesions with repeat CD.^2^, ^5^, ^6^, ^10^, ^14^ Tulandi et al.^5^ reviewed the medical records of 1283 women who underwent repeat CD and showed that increased adhesion development and a longer time to delivery were found with each subsequent CD. However, complication rates in the repeat CDs of those women were comparable with primary CD. In contrast, Silver et al.^10^ conducted a prospective observational cohort of 30 132 women who had CD, and found that blood transfusion, the duration of operative time and hospital stay significantly increased with increasing number of CD. Makoha et al.^9^ also found that morbidity increased with successive CDs before and through the third CD. Neither of those studies showed correlation between the number of CD and the occurrence of post‐CD fever, as did this study.

In addition, previous studies examined the impact of prolonged surgery duration on postoperative maternal complications,^18^, ^19^, ^20^ which were defined similarly to our study, as time exceeding the 90th percentile. Those studies found that prolonged surgery is an indicator for maternal complications such as the need for blood transfusions, prolonged hospitalization, infection necessitating antibiotics, and readmission.^18^, ^19^, ^20^ In the present study, women with severe adhesions had a longer surgery duration and a higher rate of prolonged surgery, which may explain the association between severe intra‐abdominal adhesions following CD with maternal complications that were found.

Since repeat CD correlated with maternal complications and with intra‐abdominal adhesions, it is logical to examine if severe adhesions may participate as a main cause for post‐CD complications. Taken together, these findings emphasize the importance of identifying women who are at risk for severe adhesions, given the expectation of a post‐CD course that bears a higher risk for maternal complications. Anticipating such complications following CD may enable expeditious implementation of appropriate management.

CDs are characterized by intraoperative complications primarily involving damage to adjacent organs, such as the bladder, intestines, vascular injury, significant bleeding, and infections.^21^ In this study, we examined early complications, which may be derivatives of these intraoperative complications and were thus selected for analysis. However, the study did not consider women who required a relaparotomy. Although this factor may be reflected in prolonged hospitalization and the need for blood transfusions, which are identified as complications associated with severe adhesions, it was not examined independently. Furthermore, the data was not available for the 3965 excluded due to missing data since questionnaires assessing adhesions were not filled in by the surgeons and thus could not be compared to the sample of participants included. Additionally, various factors that could influence maternal complications were not considered, such as the urgency of the surgery, prolonged rupture of membranes, blood loss estimation during the surgery, maternal complications in previous deliveries, and the results of preoperative laboratory tests. Finally, other specific morbidities that may be correlated with severe adhesions, such as bladder, bowel, and ureteric injury, were not examined in our study.

Notable advantages of the present study are its prospective design, the large sample size, the fact that the analyses were based upon multiple potential risk factors for maternal complications and the use of a validated scoring system for post‐CD adhesion assessment.^17^

## 5 CONCLUSION

This study demonstrates the significant impact of severe intra‐abdominal adhesions following CD with an increased risk of early maternal complications. These findings underscore the importance of identifying women at risk for severe adhesions that may guide efforts to reduce post‐surgical complications. Further research is required to substantiate these findings and explore measures to contain adhesion severity.

## AUTHOR CONTRIBUTIONS

**Shai Ram:** Design, planning, conduct, data analysis and manuscript writing. **Hila Shalev Ram:** Design, planning and manuscript writing. **Shira Alon:** Planning, conduct and data analysis. **Shai Trigerman:** Design, planning, conduct and manuscript writing. **Ariel Many:** Design, conduct, planning and manuscript writing. **Yariv Yogev:** Design, planning and manuscript writing. **Emmanuel Attali:** Design, planning and manuscript writing.

## FUNDING INFORMATION

None.

## CONFLICT OF INTEREST STATEMENT

The authors report no conflict of interest.

## Data Availability

Research data are not shared.
